# Development and preliminary evaluation of tongue cleaner-based pH and glucose biosensors for salivary assessment

**DOI:** 10.1016/j.jobcr.2026.101506

**Published:** 2026-08-17

**Authors:** Zoya Hussain, Shakila Mahesh

**Affiliations:** a Manav Rachna Dental College, SDS, MRIIRS, Faridabad, Haryana, India; b Department of Microbiology, Manav Rachna Dental College, SDS, MRIIRS, Faridabad, Haryana, India

**Keywords:** Salivary biosensor, Tongue cleaner, Salivary pH, Salivary glucose, Glucose oxidase biosensor

## Abstract

**Introduction:**

Saliva reflects both oral and systemic health. Monitoring salivary pH and glucose using tongue cleaner-based biosensors may provide a simple non-invasive method for routine salivary assessment.

**Objective:**

To develop and preliminarily evaluate separate tongue cleaner-based biosensors for salivary pH and glucose detection in healthy individuals and those with oral disease.

**Materials and methods:**

Separate pH and glucose biosensors were fabricated on tongue cleaner platforms. The pH biosensor used an anthocyanin–chitosan–pullulan membrane, while the glucose biosensor incorporated glucose oxidase-functionalized carbon graphite and silver/silver chloride electrodes. Eighty participants aged 18–65 years were included: 40 with dental caries or periodontitis and 40 healthy controls. The glucose sensor was calibrated using glucose standards and validated against the GOD–POD assay.

**Results:**

Salivary pH was significantly lower in the study group than controls (6.71 ± 0.41 vs 7.44 ± 0.22; p < 0.001). Glucose resistance values were significantly higher in the study group (39.03 ± 15.54 kΩ vs 10.24 ± 1.13 kΩ; p < 0.001), indicating elevated salivary glucose levels. Very large effect sizes were observed for pH, glucose resistance, and glucose levels. Potentiometric measurements strongly correlated with the GOD–POD assay (r = 0.96; p < 0.001).

**Conclusion:**

The developed tongue cleaner-based biosensors demonstrated measurable differences in salivary pH and glucose between healthy individuals and those with oral disease. These low-cost, non-invasive biosensors show potential for salivary assessment, although further large-scale validation is required.

## Graphical abstract

**Figure:**
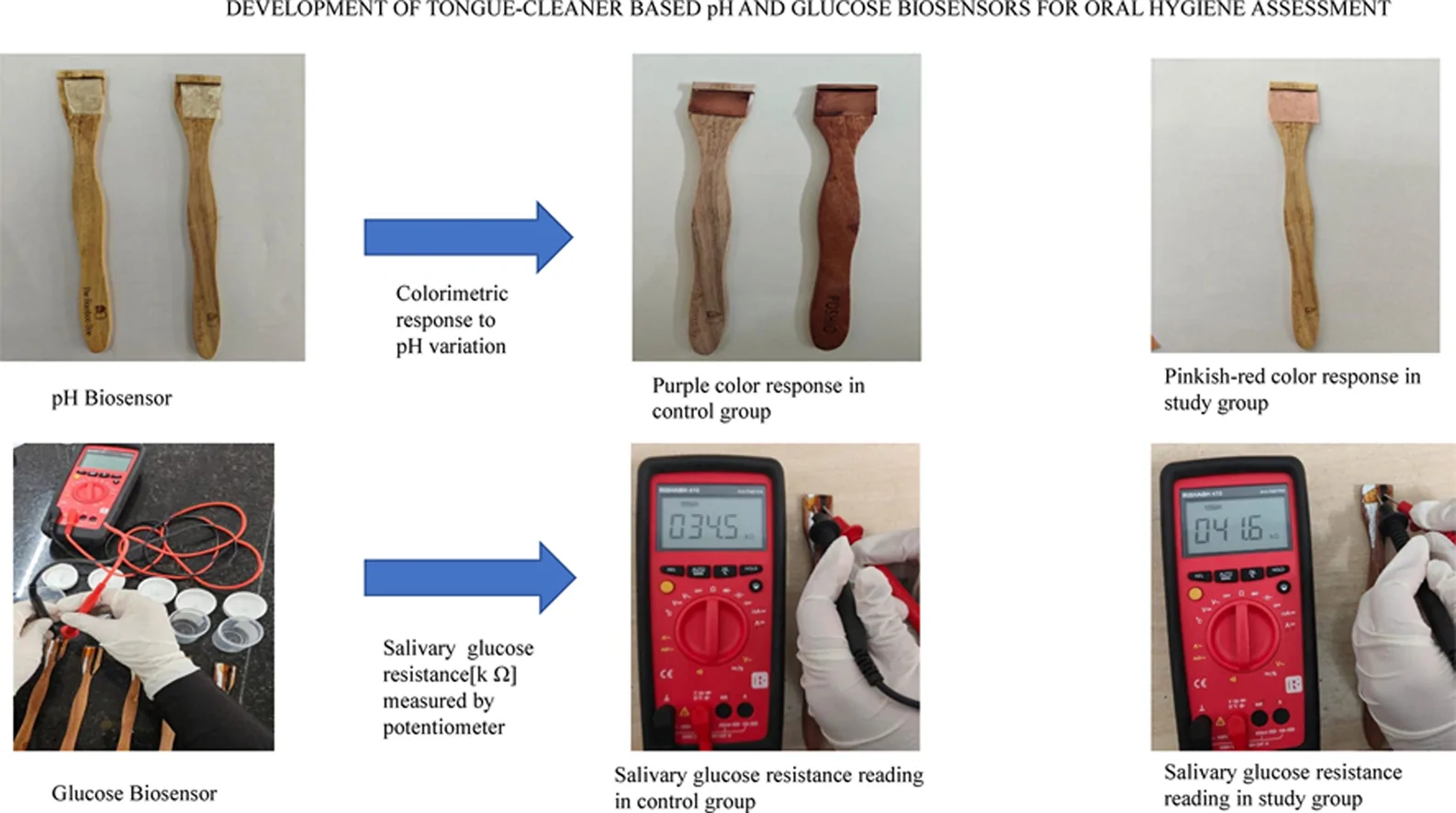

## 1 Introduction

Saliva is a valuable diagnostic fluid that reflects both oral and systemic health. It contains biomarkers such as enzymes, electrolytes, glucose, and proteins that provide important information about oral hygiene and metabolic balance. Among these, salivary pH is a key indicator of oral health, as pH fluctuations influence microbial growth, enamel demineralization, and the development of dental caries and periodontal diseases.^1^^,^^2^ Therefore, a simple and reliable method for monitoring salivary pH can support early detection of oral disorders and improve preventive care.

Naturally derived pigments such as anthocyanins have gained attention in colorimetric biosensing because of their distinct and reversible colour changes in response to pH variations. These compounds shift from red in acidic conditions to purple and blue in neutral or alkaline environments. 3, 4, 5, 6, 7 Their biocompatibility, sensitivity, and non-toxic nature make anthocyanin-based membranes suitable for oral biosensor applications.^4^

Salivary glucose measurement has also emerged as a non-invasive approach for assessing metabolic status and identifying individuals at risk of diabetes and related oral complications. Elevated salivary glucose levels may enhance microbial metabolism and acid production, resulting in lower salivary pH and thereby contributing to the development of dental caries and periodontal inflammation. 8, 9, 10 Glucose oxidase-based salivary glucose biosensors have demonstrated potential for non-invasive metabolic assessment. 11, 12, 13.

Although several salivary biosensors have been reported, many require complex instrumentation, laboratory processing, or separate sampling systems, limiting routine applicability. Integration of biosensors into commonly used oral hygiene devices remains limited. A tongue cleaner provides direct oral cavity contact, ease of handling, low manufacturing cost, and potential for incorporation into daily oral hygiene practices.^12^ Most previously reported salivary biosensors focus on a single analyte and require separate sampling or analytical devices. In contrast, the present study integrates two independent biosensors for salivary pH and glucose assessment onto a routinely used tongue-cleaner platform, providing a simple, low-cost, and non-invasive approach that may facilitate incorporation of salivary monitoring into daily oral hygiene practices.^12^

It was hypothesized that a tongue cleaner-based biosensor platform could provide measurable differences in salivary pH and glucose parameters between individuals with oral disease and healthy controls.

This study developed two independent biosensors integrated into a tongue-cleaner platform: a colorimetric anthocyanin–chitosan–pullulan membrane for pH detection and an electrochemical glucose sensor for salivary glucose measurement. Together, these components demonstrate the potential for a non-invasive and user-friendly salivary assessment platform for oral and metabolic health applications.

## 2 Materials and Methods

### 2.1 Study design and setting

**Fig. 1:**
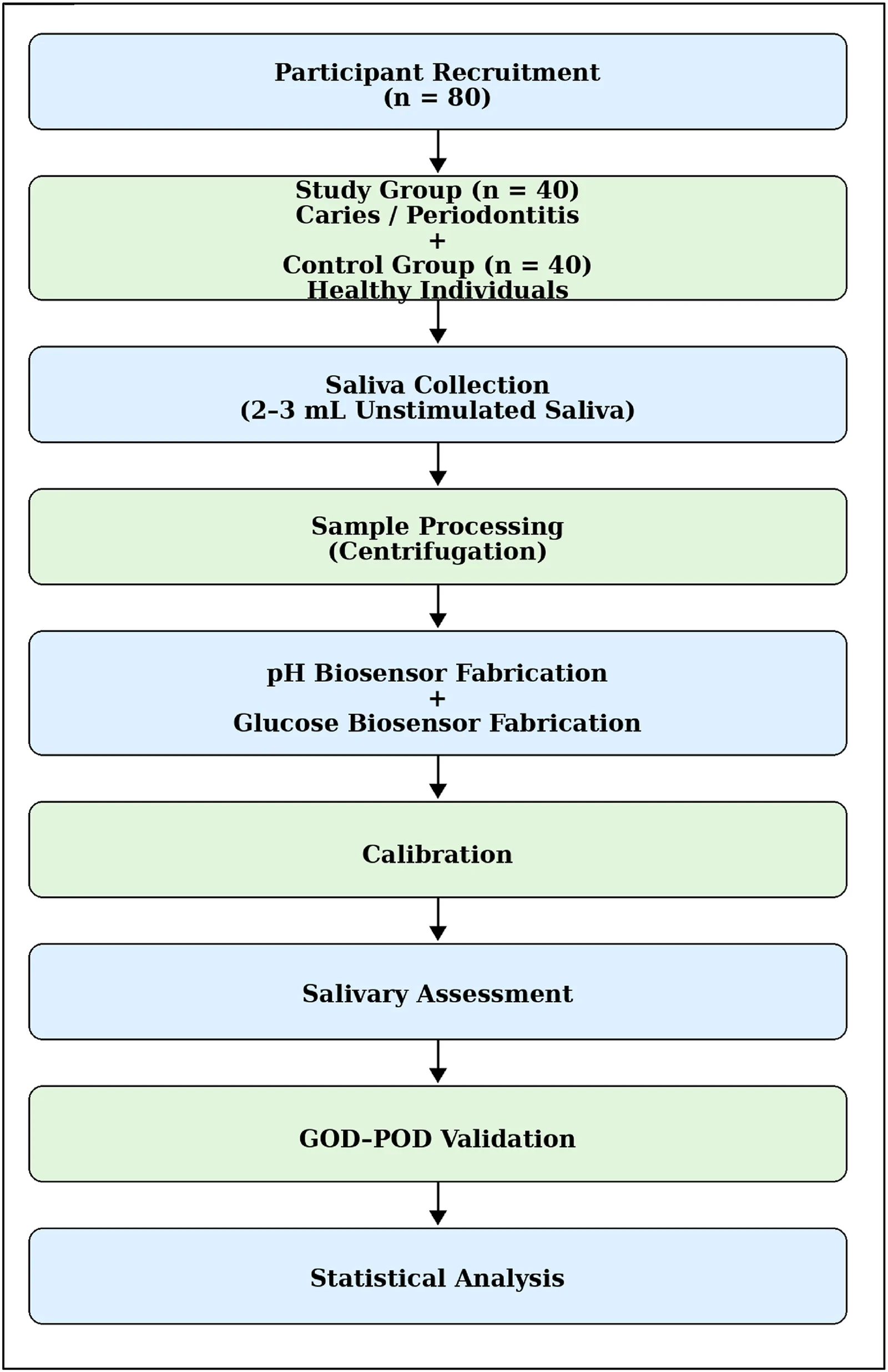
Study workflow for development and evaluation of tongue cleaner-based pH and glucose biosensors. **Footnote:** Schematic representation of participant recruitment, saliva collection, biosensor fabrication, calibration, validation, and statistical analysis.

This study followed a product process development design and was conducted in the Delhi National Capital Region over a period of one year. Ethical approval was obtained from the Institutional Ethical Committee, and written informed consent was secured from all participants before enrolment. A schematic overview of participant recruitment, saliva collection, biosensor fabrication, calibration, validation, and statistical analysis is presented in Fig. 1.

### 2.2 Study population

A total of 80 participants aged 18 to 65 years attending dental clinics for oral screening and treatment were included. Participants were divided equally into two groups: a study group consisting of individuals diagnosed with dental caries or periodontitis (n = 40) and a control group of individuals with good oral hygiene and no clinical evidence of caries or periodontal disease (n = 40).

### 2.3 Sample size determination

Sample size was calculated using G*Power software, considering an effect size of 0.8, a significance level of 5% (α = 0.05), and a study power of 80%, resulting in a total sample size of 80 participants.

### 2.4 Inclusion and exclusion criteria

Eligible participants were adults aged 18–65 years maintaining general oral hygiene practices but not regular tongue cleaning. Exclusion criteria included a history of major oral pathology or cancer, major dental treatment within the previous three months, known allergies to materials used in tongue cleaner fabrication, pregnancy, lactation, and current or recent smoking habits.

### 2.5 Specimen collection

Participants were instructed to refrain from eating, drinking (except water), chewing gum, or oral hygiene procedures for at least 1 h before saliva collection. Unstimulated whole saliva samples (2–3 mL) were collected between 10:00 a.m. and 12:00 p.m. using the passive drooling method directly into sterile graduated plastic vials without stimulation. Approximately 2–3 mL saliva was collected over 5–10 min. Participants rinsed their mouths with distilled water before collection. Samples were centrifuged at 3000 rpm for 10 min and the clear supernatant was used for testing. Following centrifugation, the clear supernatant was processed immediately for biosensor testing and GOD–POD analysis to minimize potential alterations in salivary biochemical parameters.

### 2.6 Fabrication of pH biosensor

The pH biosensor was fabricated using a biopolymer film composed of chitosan and pullulan with anthocyanin extract as the pH-sensitive dye.^4^^,^^5^^,^^14^ Chitosan and pullulan (0.25 g each) were dissolved in 50 mL of 2 M acetic acid to prepare a 0.5% (w/v) polymer solution. Anthocyanin extract (20 mg/mL) obtained from red cabbage or grape peel was added slowly under continuous stirring to achieve a homogeneous mixture. Chitosan, pullulan, acetic acid, and other chemicals used in biosensor fabrication were of analytical grade and procured from HiMedia Laboratories unless otherwise specified.

**Fig. 2:**
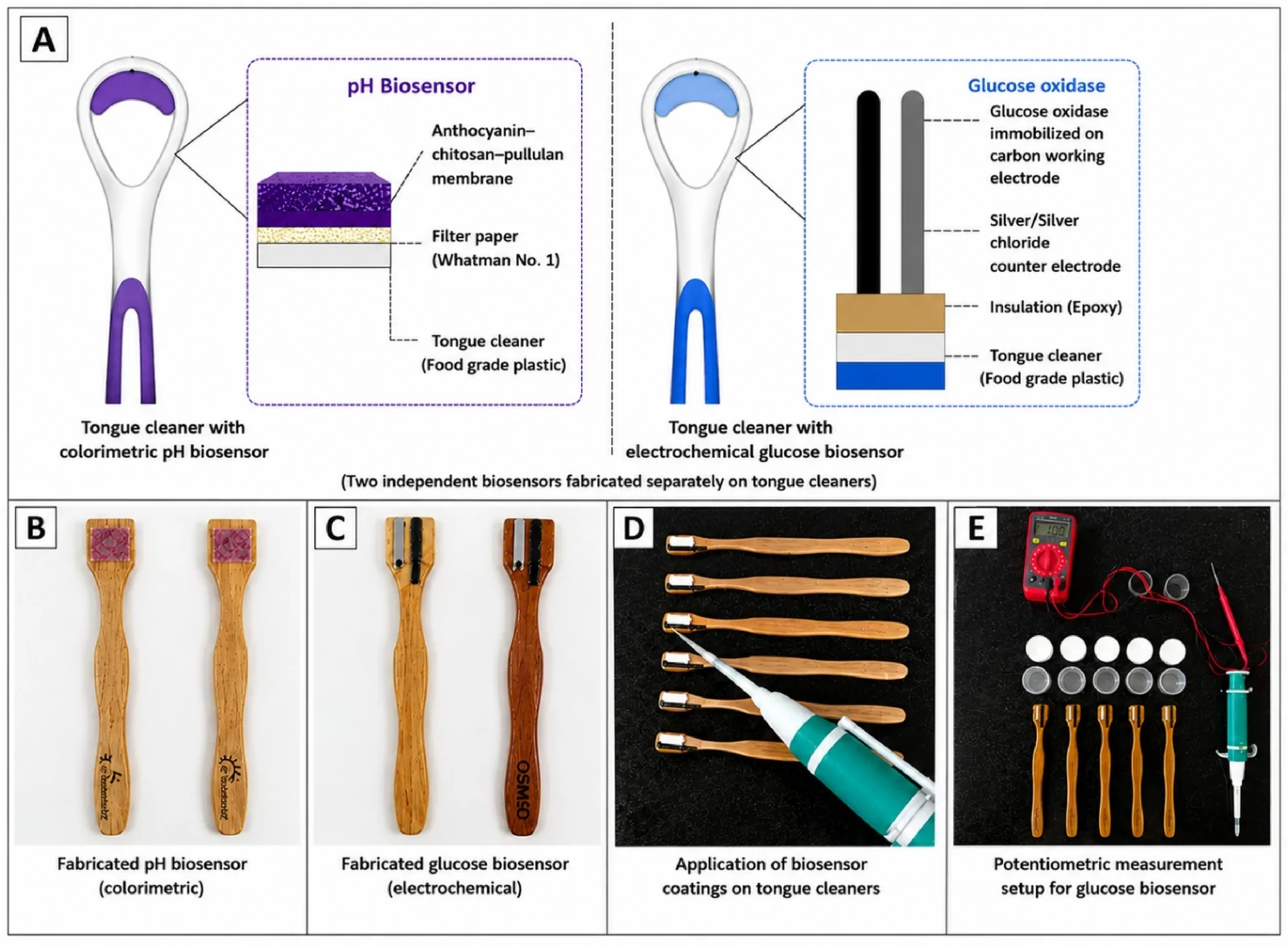
Design and fabrication of tongue cleaner-based pH and glucose biosensors. **Footnote:** (2A) Schematic representation of the pH and glucose biosensors fabricated on separate tongue cleaners. (B) Fabricated colorimetric pH biosensor. (C) Fabricated electrochemical glucose biosensor. (D) Application of biosensor coatings on tongue cleaners. (E) Potentiometric measurement setup used for glucose biosensor evaluation.

Using a micropipette, 50–100 μL of the polymer solution was spotted onto strips of Whatman No. 1 filter paper and allowed to dry at room temperature. The dried membranes were cut and affixed to the head of a food-grade tongue cleaner using a non-toxic adhesive. The fabricated pH biosensor membranes were stored in airtight sterile containers at 4°C until further use. Schematic and fabrication steps of the separate tongue cleaner-based biosensors are shown in Fig. 2.

**Fig. 3:**
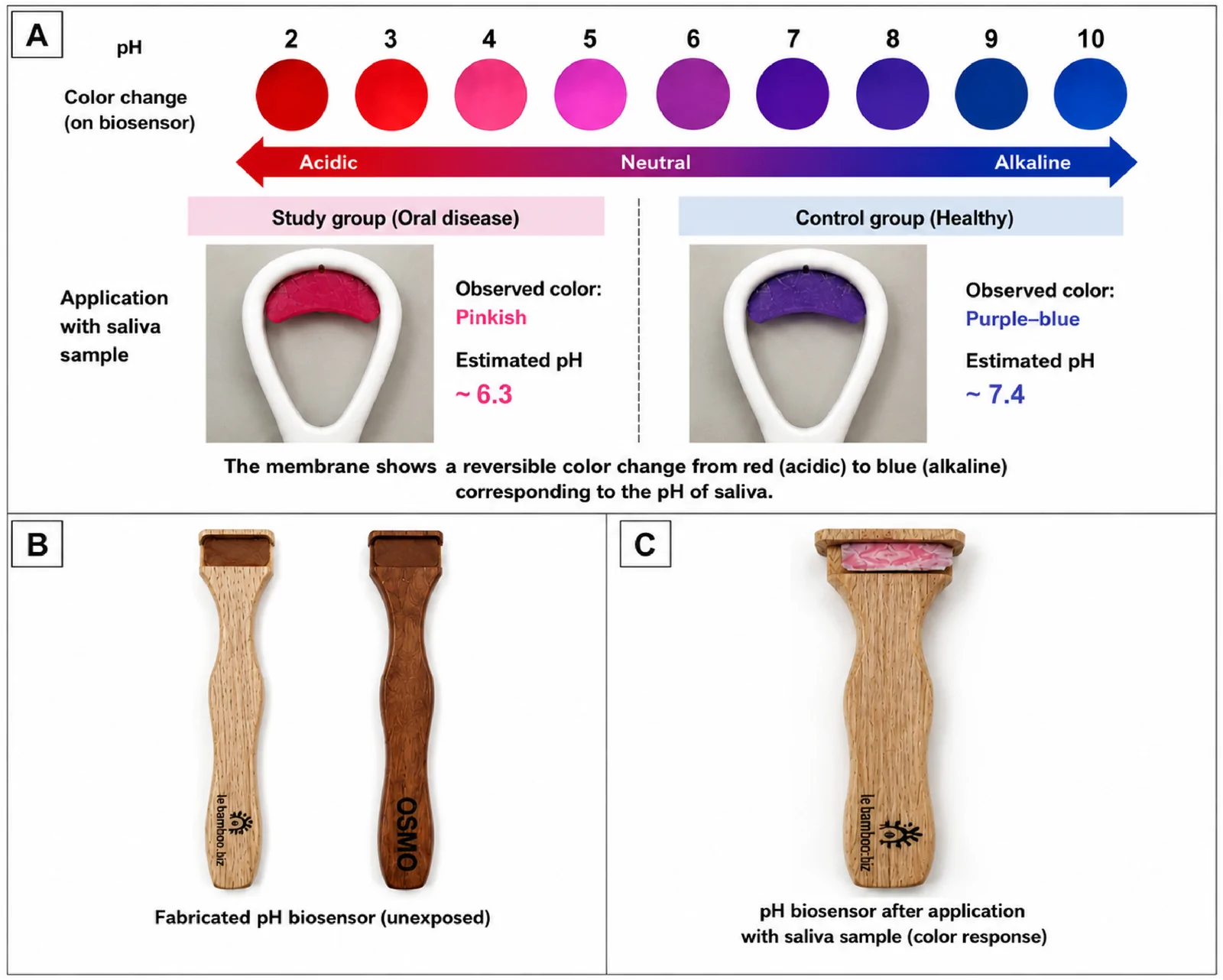
Colorimetric response and fabrication of the pH biosensor. **Footnote:** (A) Colorimetric response of the anthocyanin–chitosan–pullulan pH biosensor under acidic and neutral salivary conditions. (B) Fabricated pH biosensor before saliva application. (C) pH biosensor after saliva application demonstrating colour change corresponding to salivary pH.

The biosensor was tested by immersing the membrane in buffer solutions of varying pH (2–10) to confirm its colorimetric sensitivity and validated using a digital pH meter. The membrane exhibited reversible colour changes from red to purple to blue corresponding to acidic, neutral, and alkaline conditions respectively.^7^^,^^14^ The colorimetric response of the pH biosensor under acidic and neutral salivary conditions is illustrated in Fig. 3.

For testing, approximately 100 μL saliva was applied directly onto the biosensor membrane using a micropipette. Colour changes were visually compared with a standardized calibration chart prepared using buffer solutions (pH 2–10) and validated using a digital pH meter. The pH biosensor provided semi-quantitative colorimetric estimation rather than direct electrochemical pH measurement.

### 2.7 Fabrication of glucose biosensor

The glucose biosensor was constructed using a wooden tongue cleaner coated with conductive inks. Carbon graphite ink served as the working electrode, while silver or silver chloride ink formed the counter electrode. 10, 11, 12 The tongue cleaner surface was cleaned with 70% ethanol, air-dried, and then coated with two parallel conductive lines using a fine paintbrush. The carbon graphite working electrode and Ag/AgCl counter electrode were manually applied as two parallel strips of approximately 2–3 mm width and 12–15 mm length on the head of the wooden tongue cleaner, with an inter-electrode spacing of approximately 2 mm. As this was a prototype device, minor dimensional variation occurred during manual coating. The device was cured in a hot-air oven at 70°C for 30 min to ensure adhesion. Non-electrode areas were insulated with epoxy resin to prevent electrical interference.

After fabrication, the biosensors were stored under refrigerated conditions (4°C) prior to analysis to maintain enzyme stability. Glucose oxidase enzyme was dissolved in phosphate buffer (10 mg/mL, pH 7.4) and mixed with potassium ferricyanide as a redox mediator. All reagents used for glucose biosensor fabrication and calibration were of analytical grade. Approximately 5–10 μL of this mixture was applied onto the carbon electrode and allowed to dry at room temperature for 1 h to enable enzyme immobilization.

**Fig. 4:**
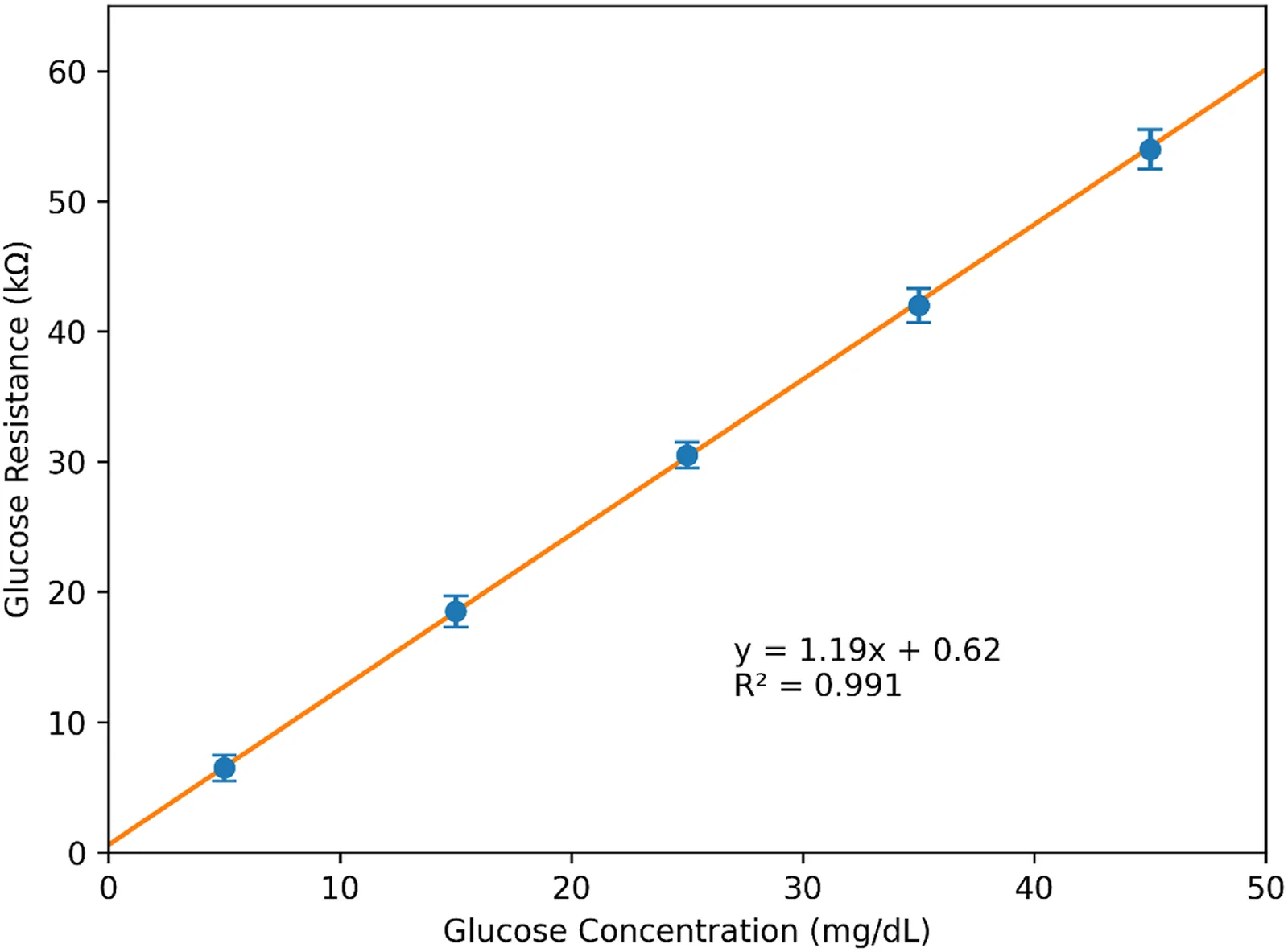
Calibration curve of the glucose biosensor. **Footnote:** Calibration of the glucose biosensor using standard glucose solutions (19–40 mg/dL). Resistance values were recorded potentiometrically and used to establish the calibration relationship.

Calibration was performed using standard glucose solutions (19, 30, and 40 mg/dL), selected to represent physiologically relevant salivary glucose ranges reported in previous studies. Each calibration measurement was performed in triplicate and mean values were used for analysis. The potentiometric response (kΩ) was recorded using a digital potentiometer. The calibration curve of the glucose biosensor is presented in Fig. 4.

### 2.8 Validation using GOD–POD endpoint method

To evaluate potentiometric measurements, the Glucose Oxidase–Peroxidase (GOD–POD) endpoint colorimetric assay was used. 15, 16, 17 In this method, glucose oxidase converts glucose to gluconic acid and hydrogen peroxide, which reacts with 4-aminoantipyrine and phenol in the presence of peroxidase to form a pink quinonimine dye measurable at 505 nm. For the GOD–POD assay, 10 μL saliva was mixed with 1 mL working reagent according to manufacturer instructions and incubated at 37°C for 15 min before absorbance measurement at 505 nm. Absorbance was measured using a spectrophotometer and all GOD–POD measurements were performed in triplicate to improve analytical reliability and minimize experimental variability.

The glucose concentration was calculated as:

Saliva Glucose (mg/dL) = Absorbance of Test × 100 Absorbance of Standard

### 2.9 Statistical analysis

Data were analyzed at a 5% significance level using Microsoft Excel, Power BI, and Python (version 3.11.4; NumPy, Pandas, SciPy). Normality was assessed using the Shapiro–Wilk test and Q–Q plots. Results are presented as mean ± standard deviation. Mann–Whitney U, Wilcoxon signed-rank, and Pearson's correlation tests were applied. Effect size was estimated using Cohen's d and interpreted according to standard thresholds. Triplicate observations were averaged before statistical analysis.

## 3 Results

A total of 80 participants were enrolled, including 40 individuals with dental caries or chronic periodontitis (study group) and 40 with good oral hygiene and no clinical signs of oral disease (control group). Gender distribution was comparable between groups (p = 0.8231), while the study group was significantly older (52.80 ± 8.16 years) than the control group (26.03 ± 5.05 years; p < 0.001), reflecting the higher prevalence of oral disease among older individuals.^1^^,^^9^ The demographic and biochemical parameters are summarized in Table 1.

**Table 1:** Demographic and salivary parameters among study and control groups.

Parameter	Study Group (n = 40)	Control Group (n = 40)	Mean Difference/Distribution	95% CI	p-value	Cohen's d
Age (years)	52.80 ± 8.16	26.03 ± 5.05	26.78	23.75 to 29.80	<0.001	—
Male, n (%)	19 (47.5)	21 (52.5)	—	—	0.8231	—
Female, n (%)	21 (52.5)	19 (47.5)	—	—	—	—
Salivary pH	6.71 ± 0.41	7.44 ± 0.22	−0.73	−0.88 to −0.58	<0.001	2.22
Glucose Resistance (kΩ)	39.03 ± 15.54	10.24 ± 1.13	28.79	23.81 to 33.77	<0.001	2.61
Salivary Glucose (mg/dL)	20.81 ± 9.93	3.03 ± 0.53	17.79	14.61 to 20.97	<0.001	2.52

Footnote: Values are expressed as mean ± standard deviation (SD) or frequency (%). P-values for gender distribution were calculated using the Chi-square test. Ninety-five percent confidence intervals (95% CI) represent confidence intervals of mean differences between groups. Cohen's d was calculated as a measure of effect size for continuous variables.

The anthocyanin–chitosan–pullulan pH biosensor showed clear and reversible colour changes corresponding to salivary pH variation, ranging from purple in neutral conditions to pinkish-red under acidic conditions. The mean salivary pH was significantly lower in the study group (6.71 ± 0.41) compared with controls (7.44 ± 0.22, p < 0.001)^2^^,^^5^^,^^17^ The observed group differences demonstrated very large effect sizes for salivary pH (Cohen's d = 2.22), glucose resistance (d = 2.61), and salivary glucose levels (d = 2.52), indicating substantial separation between study and control groups. A comparative graphical representation of the salivary parameters between study and control groups is presented in Fig. 6.

The potentiometric glucose biosensor also demonstrated consistent analytical performance. Resistance values were significantly higher in the study group (39.03 ± 15.54 kΩ) compared with the control group (10.24 ± 1.13 kΩ, p < 0.001), corresponding to elevated salivary glucose concentrations. The GOD–POD endpoint assay demonstrated strong agreement with potentiometric measurements, with mean salivary glucose levels of 20.81 ± 9.93 mg/dL in the study group and 3.03 ± 0.53 mg/dL in controls (p < 0.001). A strong positive correlation (r = 0.96, p < 0.001) was observed between potentiometric and GOD–POD readings, supporting the analytical agreement of the biosensor.^15^^,^^16^

Within the study group, participants with chronic periodontitis exhibited lower salivary pH and higher glucose resistance compared with those with dental caries. The mean pH in the periodontitis subgroup was 6.53 ± 0.20 while that of the caries subgroup was 6.97 ± 0.52. Correspondingly, glucose resistance was 48.16 ± 3.30 kΩ for periodontitis and 25.33 ± 16.65 kΩ for caries (p < 0.01). Detailed subgroup comparisons are presented in Table 2.

**Table 2:** Comparison of Salivary pH and Glucose Resistance Between Dental Caries and Chronic Periodontitis Subgroups.

Parameter	Caries (n = 20)	Periodontitis (n = 20)	Mean Difference	95% CI	p-value
Salivary pH	6.97 ± 0.52	6.53 ± 0.20	−0.44	−0.72 to −0.15	<0.01
Glucose Resistance (kΩ)	25.33 ± 16.65	48.16 ± 3.30	22.83	13.89 to 31.78	<0.01

Footnote: Values are presented as mean ± standard deviation (SD). Mean differences between dental caries and chronic periodontitis subgroups are reported with corresponding 95% confidence intervals (CI). Statistical significance was assessed at p < 0.05.

**Fig. 5:**
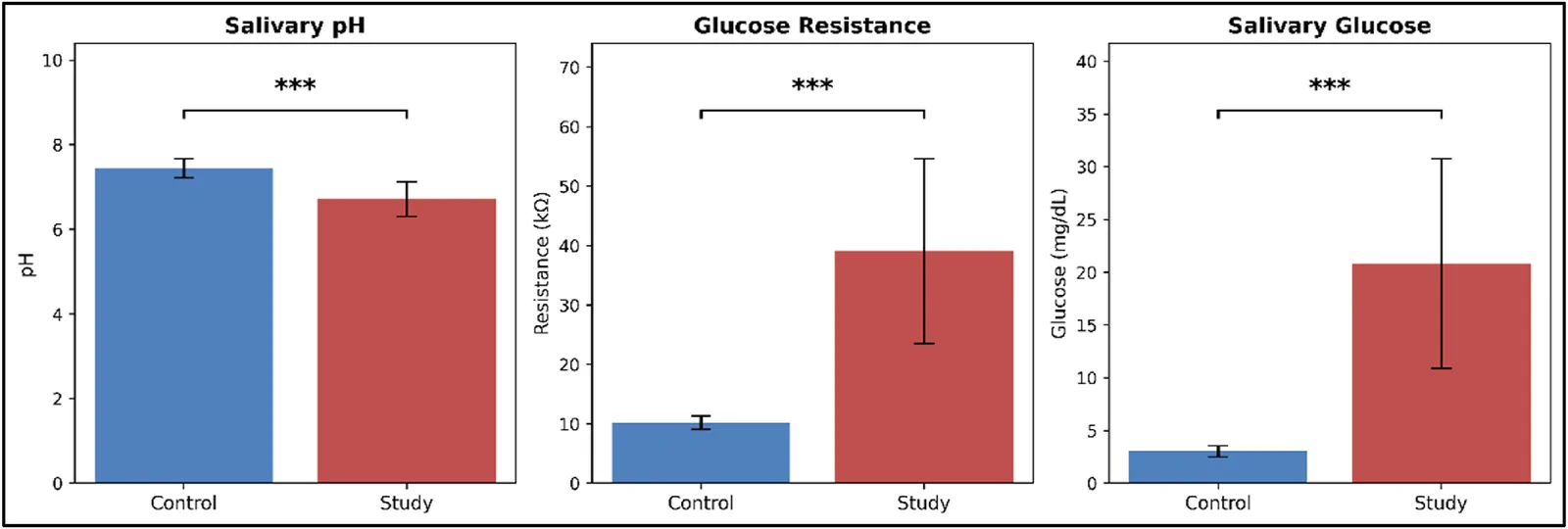
Comparison of salivary parameters between study and control groups. **Footnote:** Comparative analysis of salivary pH, glucose resistance, and salivary glucose levels between participants with oral disease (study group) and healthy controls. Error bars represent standard deviation. ***p < 0.001.

A significant inverse correlation was observed between salivary pH and glucose resistance (r = −0.62; 95% CI: −0.77 to −0.35; p < 0.001), indicating that increased glucose concentration was associated with greater acidity, supporting the link between carbohydrate metabolism and acidogenesis in oral disease.^9^^,^^18^^,^^19^ Detailed correlation analysis is presented in Table 3. A graphical representation of the correlation between salivary pH and glucose resistance is presented in Fig. 5.

**Table 3:** Correlation analysis within study group.

Variables	Pearson's r	95% CI	p-value
Salivary pH vs Glucose Resistance	−0.62	−0.77 to −0.35	<0.001

Footnote: Pearson's correlation coefficient (r) is presented with corresponding 95% confidence intervals (CI) and p-values. A negative correlation indicates that higher glucose resistance values were associated with lower salivary pH within the study group.

**Fig. 6:**
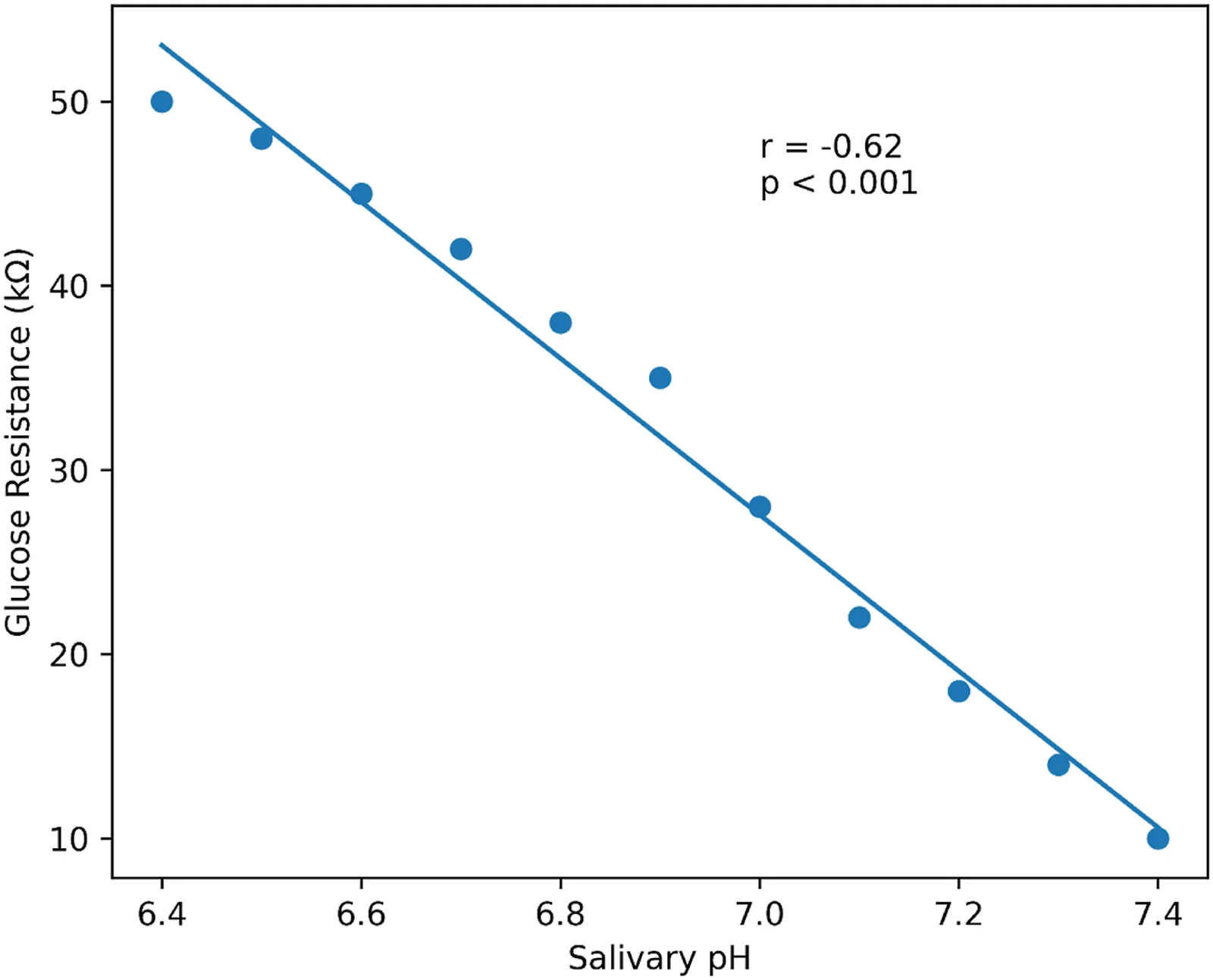
Correlation between salivary pH and glucose resistance in the study group. **Footnote:** Scatter plot showing the inverse relationship between salivary pH and glucose resistance measured using the biosensor platform. Pearson correlation coefficient and regression line are shown.

Both biosensors integrated onto the tongue-cleaner platform demonstrated consistent analytical performance during preliminary evaluation. The colorimetric pH biosensor allowed immediate visual interpretation, and the glucose sensor provided quantitative potentiometric readings demonstrating strong agreement with the GOD–POD assay. Together, these independent biosensors demonstrate the potential for non-invasive salivary assessment of pH and glucose on a tongue-cleaner platform (Table 1, Table 2).

## 4 Discussion

This study demonstrated the successful development of separate pH and glucose biosensors integrated onto a tongue-cleaner platform for preliminary non-invasive salivary assessment. The anthocyanin–chitosan–pullulan membrane showed consistent colorimetric responsiveness to salivary acidity, while the glucose oxidase–based potentiometric sensor demonstrated strong agreement with the conventional GOD–POD assay. Individuals with dental caries or periodontitis exhibited significantly lower salivary pH and higher glucose-related measurements compared with healthy controls (Table 1), with very large effect sizes observed for all major salivary parameters, supporting the magnitude of group differences. These findings are consistent with previous studies reporting altered salivary biochemical profiles in oral disease conditions.^1^^,^^5^^,^^9^^,^^16^

Salivary pH is widely recognized as an important indicator of oral health because acidic conditions promote enamel demineralization, microbial acidogenesis, and periodontal inflammation.^2^^,^^5^^,^^17^ In the present study, the diseased group demonstrated significantly lower salivary pH values than controls, aligning with observations reported by Baliga et al. and Narasimman et al.^5^^,^^17^ The reversible colour transition of the anthocyanin membrane from acidic to alkaline conditions also supports the optical stability and applicability of naturally derived pigments in oral biosensing systems, while previous studies have also reported that anthocyanin behaviour may be influenced by surrounding glucose concentration and pH conditions.^7^^,^^20^^,^^21^ Integration of the membrane onto a tongue cleaner further enhances its practicality by enabling rapid visual assessment during routine oral hygiene practices without requiring sophisticated instrumentation.

The glucose biosensor effectively differentiated study participants from controls and showed strong correlation with GOD–POD measurements (r = 0.96, p < 0.001), indicating promising analytical agreement. Similar associations between elevated salivary glucose levels, periodontal disease, and metabolic alterations have been described previously.^6^^,^^8^, ^9^, ^10^, ^11^^,^^15^ Elevated salivary glucose may enhance microbial growth and acid production, thereby contributing to progression of oral disease. Compared with previously reported saliva-based biosensors and emerging non-invasive saliva glucose monitoring systems, the present platform combines assessment of two clinically relevant salivary parameters using separate biosensors integrated onto a commonly used oral hygiene device. This design may improve practicality and user acceptability while maintaining a simple and low-cost fabrication approach.^12^^,^^22^^,^^23^ The observed inverse correlation between salivary pH and glucose resistance (r = −0.62, p < 0.001) further supports the biochemical relationship between carbohydrate metabolism and oral acidogenic activity, consistent with earlier investigations.^9^^,^^18^^,^^19^

Overall, the findings reinforce the potential utility of saliva as a clinically relevant biofluid and support growing interest in saliva-based point-of-care diagnostic technologies for oral and systemic health assessment.^24^^,^^25^ Although the study group was significantly older than the control group, this difference reflects the natural clinical distribution of chronic periodontitis, which is more commonly encountered in older individuals and is frequently associated with metabolic alterations. Nevertheless, age may still represent a confounding factor and future studies should incorporate age-matched cohorts and multivariate-adjusted analyses. However, larger longitudinal studies and comprehensive analytical validation are necessary before broader clinical translation.

## 5 Limitations

This study has certain limitations. The cross-sectional design restricts causal interpretation between salivary biochemical alterations and oral disease progression. The sample size, although adequate for preliminary proof-of-concept evaluation, was relatively small for broader generalization. In addition, convenience sampling was used in this clinic-based study, which may limit external validity. A significant age imbalance was present between the study and control groups, and age may have influenced salivary biochemical parameters independently of oral disease status. Therefore, the observed differences in salivary pH and glucose-related measurements should be interpreted with caution. The colorimetric pH biosensor provided semi-quantitative estimation and may be influenced by subjective visual interpretation. In the glucose biosensor, enzyme immobilization was achieved through drop-casting, which may affect long-term stability and reproducibility. Furthermore, sensitivity, specificity, long-term storage stability, and broader clinical robustness of the biosensors were not comprehensively evaluated. Ongoing and future studies with larger sample sizes, age-matched cohorts, longitudinal follow-up, and multivariate-adjusted analyses are planned to further validate the findings and enhance the diagnostic utility of the proposed biosensor platform.

## 6 Conclusion

The present proof-of-concept study demonstrated that tongue cleaner-based pH and glucose biosensors could detect measurable differences in salivary parameters between healthy individuals and those with oral disease. The anthocyanin–chitosan–pullulan membrane showed consistent response to salivary pH variation, while the glucose oxidase-based potentiometric sensor demonstrated promising agreement with the GOD–POD method. Participants with oral disease exhibited lower salivary pH and higher glucose-related measurements, supporting the association between salivary biochemical alterations and oral disease status. The developed platform represents a simple, low-cost, and non-invasive approach with potential applicability in routine oral hygiene assessment. However, further large-scale validation, age-matched studies, and long-term analytical evaluation are required before broader clinical application.

## Patient's consent

Prior to collection of saliva in the plastic vials, the written consent form was signed by the patient in full awareness of the protocol of the study.

## Informed consent

Written informed consent was obtained from all participants prior to sample collection.

## Ethical clearance

Ethical approval was sought from Institutional Ethical Committee (code- 2024/84) dated 24 July 2024.

## Author contribution

Zoya Hussain- Conceptualization, Data curation, Methodology, Investigation, Visualization.

Dr. Shakila Mahesh- Writing-Original Draft, Validation, Super-vision Writing- Review and editing.

## Ethical approval

The study protocol was approved by the Institutional Ethics Committee of Manav Rachna Dental College, School of Dental Sciences, Manav Rachna International Institute of Research and Studies (MRDC/IEC/2024/84).

## Human and animal rights statement

All procedures performed in this study involving human participants were conducted in accordance with the ethical standards of the Institutional Ethics Committee of Manav Rachna Dental College, Faridabad, India, and with the 1964 Declaration of Helsinki and its later amendments. Ethical approval was obtained from the Institutional Ethics Committee (Approval No.: (MRDC/IEC/2024/84).

No animals were used in this study.

## Funding information

ICMR STS approved study and stipend of 30000 was provided to student after successful submission of the report.

## Declaration of competing interest

The authors declare that they have no known competing financial interests or personal relationships that could have appeared to influence the work reported in this paper.
